# Effects of non-invasive ventilation and posture on chest wall volumes and motion in patients with amyotrophic lateral sclerosis: a case series

**DOI:** 10.1590/bjpt-rbf.2014.0164

**Published:** 2016-06-16

**Authors:** Cristiana M. Magalhães, Guilherme A. Fregonezi, Mauro Vidigal-Lopes, Bruna S. P. P. Vieira, Danielle S. R. Vieira, Verônica F. Parreira

**Affiliations:** 1 Programa de Pós-graduação em Ciências da Reabilitação, Universidade Federal de Minas Gerais (UFMG), Belo Horizonte, MG, Brazil; 2 Laboratório de Desempenho PneumoCardioVascular e Músculos Respiratórios, Departamento de Fisioterapia, Universidade Federal do Rio Grande do Norte (UFRN), Natal, RN, Brazil; 3 PneumoCardioVascular Lab, Hospital Universitário Onofre Lopes, Empresa Brasileira de Serviços Hospitalares (EBSERH), UFRN, Natal, RN, Brazil; 4 Programa Vent-Lar - Ventilação Mecânica Domiciliar para Pessoas com Doenças Neuromusculares do Estado de Minas Gerais, Serviço de Pneumologia, Hospital Júlia Kubitschek, Fundação Hospitalar do Estado de Minas Gerais – FHEMIG, Belo Horizonte, MG, Brazil; 5 Hospital das Clínicas, UFMG, Belo Horizonte, MG, Brazil; 6 Curso de Fisioterapia, Universidade Federal de Santa Catarina (UFSC), Araranguá, SC, Brazil; 7 Departamento de Fisioterapia, UFMG, Belo Horizonte, MG, Brazil

**Keywords:** amyotrophic lateral sclerosis, neuromuscular diseases, lung volume measurements, non-invasive ventilation, rehabilitation

## Abstract

**Background:**

The effects of non-invasive ventilation (NIV) on the breathing pattern and thoracoabdominal motion of patients with amyotrophic lateral sclerosis (ALS) are unknown.

**Objectives:**

1) To analyze the influence of NIV on chest wall volumes and motion assessed by optoelectronic plethysmography in ALS patients and 2) to compare these parameters in the supine and sitting positions to those of healthy individuals (without NIV).

**Method:**

Nine ALS patients were evaluated in the supine position using NIV. In addition, the ALS patients and nine healthy individuals were evaluated in both sitting and supine positions. Statistical analysis was performed using the paired Student t-test or Wilcoxon test and the Student t-test for independent samples or Mann-Whitney U test.

**Results:**

Chest wall volume increased significantly with NIV, mean volume=0.43 (SD=0.16)L versus 0.57 (SD=0.19)L (p=0.04). No significant changes were observed for the pulmonary rib cage, abdominal rib cage, or abdominal contribution. The index of the shortening velocity of the diaphragmatic muscle, mean=0.15 (SD=0.05)L/s versus 0.21 (SD=0.05)L/s (p<0.01), and abdominal muscles, mean=0.09 (SD=0.02)L/s versus 0.14 (SD=0.06)L/s (p<0.01), increased during NIV. Comparisons between the supine and sitting positions showed similar changes in chest wall motion in both groups. However, the ALS patients presented a significantly lower contribution of the abdomen in the supine position compared with the controls, mean=56 (SD=13) versus 69 (SD=10) (p=0.02).

**Conclusions:**

NIV improved chest wall volumes without changing the contribution of the chest wall compartment in ALS patients. In the supine position, ALS patients had a lower contribution of the abdomen, which may indicate early diaphragmatic dysfunction.

## BULLET POINTS

- Non-invasive ventilation led to a statistically significant increase in chest wall tidal volume, end-inspiratory volume, and end-expiratory volume, as well as minute ventilation, which could contribute to the preservation of the peripheral airway patency and better hematosis.
- Despite the increase in chest wall volumes, no changes were observed in the compartmental distributions of the volume during non-invasive ventilation.
- Patients with ALS had a statistically significant lower contribution of the abdomen compared with the control group when evaluated in the supine position.
- Optoelectronic plethysmography was able to reveal modifications in the breathing pattern related to ALS even in early-stage patients.

## Introduction

The reduction in lung volumes combined with respiratory muscle weakness in patients with amyotrophic lateral sclerosis (ALS) is responsible for chronic hypoventilation, which can cause major disorders, particularly those related to sleep^1^, and can contribute to respiratory insufficiency, the major cause of death in ALS patients^2^. Non-invasive ventilation (NIV) is an effective therapeutic procedure that improves the ventilation/perfusion relationship and is widely used in patients with ALS^3^ ^,^ ^4^. However, the possible physiological responses that this treatment can produce in terms of respiratory muscle action and chest wall mechanics have not yet been identified.

Different measures have been proposed for the assessment of diaphragmatic dysfunction in this population, including comparisons of the forced vital capacity in the supine and standing positions, changes in blood gases, symptoms and respiratory muscle strength^5^. However, there has been no method considered to be the gold standard^3^. Optoelectronic plethysmography (OEP) is a method recently proposed to measure chest wall volumes. OEP enables the assessment of the contribution of the three compartments of the chest wall (pulmonary rib cage, abdominal rib cage, and abdomen) and the assessment of different intervention methods using a range of respiratory rehabilitation techniques. Variables from OEP can also be used to estimate the degree of diaphragm participation^6^. More specifically, it can be used to calculate its shortening velocity^7^ and to assess the effect that positioning has on the performance of this muscle^8^.

Some authors have already assessed patients with neuromuscular diseases (NMDs) using OEP. Lo Mauro et al.^9^ studied the contribution of chest wall compartments in the supine and sitting positions in patients with Duchenne muscular dystrophy (DMD) and observed a volume reduction of the abdominal compartment in the supine position as the disease progressed. D’Angelo et al.^10^ studied patients with slow-course muscular dystrophies and concluded that OEP was useful to detect the involvement of the respiratory muscles in the initial stages of the disease. This could prove valuable in the evaluation of respiratory function, as well as the response to therapeutic strategies.

To our knowledge, this is the first study to assess the effects of NIV on chest wall volumes and motion in patients with ALS. The investigation of these parameters can provide information about the mechanisms involved in the improvement of ventilation during NIV in these patients. Moreover, the tri-compartmental analysis that can be performed using OEP can help to detect modifications in the breathing pattern related to ALS even in early-stage patients.

The primary objective of this study was to assess the effects of NIV on the volumes and motion of the chest wall and its compartments in patients with ALS. The secondary objective was to analyze these parameters, without NIV, in the supine and sitting positions in patients with ALS and to compare these with values from age- and sex-matched healthy individuals.

## Method

### Study design and participants

This was a cross-sectional study (case series). Patients diagnosed with ALS and healthy individuals were studied. The inclusion criteria for the ALS group were a diagnosis of ALS according to *El Escorial* criteria^11^, non-smokers, no scoliosis or chest abnormalities, no clinical signs of bulbar muscular dysfunction^12^, no history of tracheostomy, and the ability to complete the tests without the use of NIV. The control group included healthy age- and sex-matched individuals with a body mass index (BMI) that did not classify them as underweight (<18.5 kg/m^2^) or obese (≥30 kg/m^2^)^13^, without scoliosis or chest abnormalities, without smoking habit, and with normal spirometry findings according to the reference values described by Pereira et al.^14^. The exclusion criterion considered for both groups was the inability to complete any of the test procedures. This study was approved by the Ethics Committee of Universidade Federal de Minas Gerais (UFMG), Belo Horizonte, MG, Brazil (ETIC 0395.0.203.000-10), and all participants provided written consent.

### Procedures

The assessments were conducted over two days within a two-week period. On the first day, an initial assessment of the participants was performed for identification, characterization, and verification of the inclusion and exclusion criteria. For both groups, we measured height, body mass (Filizola Ind. São Paulo, SP, Brazil), arterial blood pressure (BD sphygmomanometer; Becton, Dickinson and Company, Franklin Lakes, NJ, USA; LittmannClassic II stethoscope; 3M Center, St. Paul, MN, USA), peripheral oxygen saturation and heart rate (Datex-Ohmeda TuffSat®; GE Healthcare Finland Oy, Helsinki, Finland). In addition, we measured peak expiratory flow and cough peak flow (Asmaplan+; Vitalograph, Ennis, Ireland) and conducted a sniff test (digital manovacuometer; NEPEB-LabCare/UFMG, Belo Horizonte, MG, Brazil) and a spirometry test (Pony FX; Cosmed srl, Rome, Italy).

In the patients with ALS, a maximum insufflation capacity (MIC) test was also conducted for classification in terms of the presence or absence of bulbar muscle dysfunction^15^, which could be a source of bias. The MIC was obtained by air stacking delivered via an oronasal mask from a manual resuscitator. When the MIC was equal to the FVC, it was considered to indicate the presence of bulbar muscle dysfunction. The Amyotrophic Lateral Sclerosis Functional Rating Scale-Revised (ALSFRS-R/BR)^16^ and the Amyotrophic Lateral Sclerosis Assessment Questionnaire (ALSAQ-40/BR)^17^ were also administered. The ALSFRS-R/BR is scored from 0–48, where a lower score indicates worse function, while the ALSAQ-40/BR has scores of 0–100, in which higher scores indicate a worse quality of life.

On the second day, the chest wall movement was assessed in the sitting and supine positions, both at rest. The individuals were positioned in a sitting position on a standardized seat without trunk support, with their upper limbs and shoulders abducted while comfortably supported. Measurements were conducted with the patient in the sitting position over a period of five minutes. Subsequently, the participants were assessed in the supine position and the chest wall volumes were evaluated over five minutes. For the participants in the ALS group, the analysis was also conducted in the supine position for five minutes during NIV (Trilogy 100; Respironics, Murrysville, PA, USA) using a nasal or face mask (Easy Life or Spectrum, respectively; Respironics, Murrysville, PA, USA), which was selected after considering the patients’ comfort^18^ to properly adjust the NIV. The spontaneous/timed mode was used with an inspiratory positive airway pressure of 14 cmH_2_O and an expiratory positive airway pressure of 7 cmH_2_O^18^, with a backup respiratory frequency of 14 bpm and an inspiratory time of one second.

### Measurement instruments

Optoelectronic plethysmography (BTS Bioengineering, Milan, Italy) is capable of assessing breath-by-breath changes in the total volume of the chest wall and the contributions of its three compartments (pulmonary rib cage, abdominal rib cage, and abdomen)^8^ ^,^ ^19^. The OEP instrument consists of a system that focuses on motion analysis and is composed of video cameras that emit an infrared light beam that is reflected by the markers and captured by the cameras^19^ ^,^ ^20^. The measurement properties, operation principles, calibration procedure, and protocol for positioning the 89 reflective markers in the sitting position and 52 markers in the supine position for the analysis were described previously^7^ ^,^ ^19^ ^,^ ^21^. Measurements of the peripheral oxygen saturation and heart rate were continually conducted throughout data collection for monitoring purposes using pulse oximetry (Datex-Ohmeda TuffSat®; GE Healthcare Finland Oy, Helsinki, Finland).

### Variables analyzed

The following primary variables were analyzed: tidal volume of the chest wall (V_cw_), percentage of the contribution of the pulmonary rib cage (V_rcp_%), percentage of the contribution of the abdominal rib cage (V_rca_%), percentage of the contribution of the abdomen (V_ab_%), chest wall end-inspiratory volume, and chest wall end-expiratory volume. The secondary variables analyzed included respiratory rate (*f*), minute ventilation (VE), ratio of inspiratory time to total duration of respiratory cycle (Ti/Ttot), the phase angle (PhAng), phase relation during inspiration (PhRIB), and phase relation during expiration (PhREB). The MATLAB® software program was used to analyze the thoracoabdominal asynchrony. Given that the changes in diaphragm muscle fiber length can be estimated by determining the ratio between the abdominal volume variation and the inspiratory time, this ratio was used as an index of the shortening velocity of the diaphragm muscle^7^. The index of shortening velocity of the inspiratory muscles of the pulmonary rib cage and the index of the shortening velocity of the abdominal muscles were also calculated^22^.

### Statistical analysis

A sample size calculation was performed based on a size effect calculation using the group means and their common standard deviation of the Vcw (a main outcome variable) in the supine position with and without NIV for the patients with ALS (Vcw without NIV: 0.43±0.16 L, Vcw with NIV: 0.57±0.19 L); considering a power of 80% and a significance level of 5%^23^. The sample obtained was of nine subjscts. The normality of data was verified using the Shapiro-Wilk test. A comparison between the data from the ALS group and the control group (anthropometric and kinematic data in the supine position without NIV and in the sitting position) was conducted using Student’s t-test for independent samples or the Mann-Whitney U test, as appropriate. To perform the between-group comparison of the controls (in a seated or supine position) and the ALS group (supine position with or without NIV), the paired Student’s t-test or Wilcoxon test were used. The data were analyzed using the Statistical Package for Social Sciences software program version 15.0 (SPSS Inc., Chicago, IL, USA).

## Results

Seventeen patients with ALS were recruited for this study. Four of these patients were ineligible due to the presence of bulbar dysfunction, and another four were excluded: two after refusing to participate, one due to transportation difficulties, and the other for being unable to maintain a sitting position without using the back support. Overall, nine patients completed the study protocol (eight patients used a face mask during NIV), along with nine age- and sex-matched healthy individuals.

Table 1 presents the participants’ demographic and anthropometric characteristics, pulmonary function, functionality, and quality of life data for the patients with ALS and controls. The BMI, forced vital capacity, forced expiratory volume in one second, sniff test, cough peak flow, and peak expiratory flow values were significantly lower in the patients with ALS than in the controls. No significant differences were observed among the remaining variables.

**Table 1:** The participants’ demographic and anthropometric characteristics, pulmonary function, functionality and quality of life data in patients with ALS and controls.

**VARIABLES**	**ALS group**	**Control group**	**p value**
**Sex (Male/Female)**	5/4	5/4	-
**Age (years)**	55 (13)	55 (15)	0.97
**BMI (Kg/m^2^)**	21 (4)	24 (2)	0.04^*^
**FVC (%predicted)**	56 (18)	95 (10)	<0.01^*^
**FEV_1_ (%predicted)**	57 (17)	95 (10)	<0.01^*^
**FEV_1_/FVC (%)**	84 (9)	80 (4)	0.28
**MIC (%predicted)**	62 (14)	-	-
**SNIP (cmH_2_O)**	41 (25)	73 (11)	<0.01^*^
**CPF (L/min)**	232 (111)	478 (146)	<0.01^*^
**PEF (L/min)**	246 (81)	468 (149)	<0.01^*^
**ALSFRS-R/BR (0-48)**	32 (8)	-	-
**ALSAQ-40/BR (0-100)**	49 (17)	-	-

The data are presented as the means and standard deviations. ALS: amyotrophic lateral sclerosis; BMI: body mass index; FVC: forced vital capacity; FEV_1_: forced expiratory volume in one second; FEV_1_/FVC: ratio of the forced expiratory volume in one second to the forced vital capacity; MIC: maximum insufflation capacity; SNIP: sniff nasal inspiratory pressure; CPF: cough peak flow; PEF: peak expiratory flow; ALSFRS-R/BR: Amyotrophic Lateral Sclerosis Functional Rating Scale- Revised; ALSAQ-40/BR: Amyotrophic Lateral Sclerosis Assessment Questionnaire. Student’s t-test (independent samples) or *the* Mann-Whitney U test (FVC) was used for the statistical analyses.

* p<0.05 for the comparison of the ALS and control groups.

Table 2 shows the chest wall volumes, contributions of chest wall compartments, and breathing time variables in patients with ALS with and without NIV. A significant increase in the V_cw_, chest wall end-inspiratory volume, chest wall end-expiratory volume, and VE was observed with the use of NIV. There was a mean increase of 0.58 L in the chest wall end-inspiratory volume, 0.43 L in the chest wall end-expiratory volume and 0.14 L in the V_cw_ due to NIV use. 

**Table 2:** The chest wall volumes, contributions of the chest wall compartments and respiratory time variables in patients with ALS with and without non-invasive ventilation.

**VARIABLES**	**ALS without NIV**	**ALS with NIV**	**Between-group** **difference**	**95% CI**	**p value**
**V_cw_ (L)**	0.43 (0.16)	0.57 (0.19)	0.14	0.01 to 0.27	0.04^*^
**V_rcp_%**	32 (13)	34 (15)	3	–11 to 16	0.67
**V_rca_%**	13 (5)	12 (6)	–1	–4 to 1	0.30
**V_ab_%**	56 (13)	54 (17)	–1	–16 to 14	0.87
**Vei_cw_ (L)**	18.37 (3.84)	18.95 (3.85)	0.58	0.34 to 0.81	<0.01^*^
**Vee_cw_ (L)**	17.94 (3.85)	18.37 (3.94)	0.43	0.26 to 0.60	<0.01^*^
**VE (L/min)**	6.44 (2.07)	10.13 (3.77)	3.69	0.50 to 6.88	0.03^*^
***f* (bpm)**	16 (5)	18 (7)	---	–3 to 8	0.59
**Ti/Ttot**	0.38 (0.04)	0.39 (0.08)	0.07	–0.05 to 0.06	0.77

The data are presented as the means and standard deviations. 95% CI: 95% confidence interval; ALS: amyotrophic lateral sclerosis; NIV: non-invasive ventilation; V_cw_: chest wall volume; V_rcp_%: percentage of the contribution of the pulmonary rib cage; V_rca_%: percentage of the contribution of the abdominal rib cage; V_ab_%: percentage of the contribution of the abdomen; Vei_cw_: chest wall end-inspiratory volume; Vee_cw_: chest wall end-expiratory volume; VE: minute ventilation *f*: respiratory rate; Ti/Ttot: ratio of the inspiratory time to the total duration of the respiratory cycle. Student’s paired t-test or the Wilcoxon (*f*) test was used.

* p<0.05 for comparisons of the ALS groups with and without NIV.

Table 3 shows the chest wall volumes, contributions of the chest wall compartments, and breathing time variables in the sitting and supine positions for the ALS patients and control group. The V_rcp_% and V_rca_% were significantly lower, while the V_ab_% was significantly higher, in the supine position compared with the sitting position in both groups. Significantly higher V_rca_% values and lower V_ab_% values were observed in the supine position and higher *f* and lower V_cw_ values were observed in the sitting position in the ALS group than in the control group.

**Table 3:** The chest wall volumes, contributions of the chest wall compartments and respiratory time variables in the sitting and supine positions in patients with ALS and controls.

**VARIABLES**	**ALS group**					**Control group**					
	**Sitting**	**Supine**	**Between-group** **difference**	**95% CI**	**p-value**		**Sitting**	**Supine**	**Between-group** **difference**	**95% CI**	**p-value**
**V_cw_ (L)**	0.45 (0.13)^†^	0.43 (0.16)	---	–0.11 to 0.12	0.51		0.57 (0.13)	0.51 (0.18)	--	–0.04 to 0.18	0.21
**V_rcp_%**	40 (19)	32 (13)	9	1 to 19	0.04		41 (6)	23 (10)	18	13 to 23	<0.01
**V_rca_%**	19 (9)	13 (5)^*^	6	1 to 12	0.04		20 (5)	8 (2)	13	10 to 15	<0.01
**V_ab_%**	41 (16)	56 (13)^*^	-15	–23 to –7	<0.01		39 (9)	69 (10)	–30	–35 to -26	<0.01
**Vei_cw_ (L)**	21.26 (6.75)	18.37 (3.84)	2.89	–2.31 to 8.09	0.24		22.81 (6.24)	19.34 (3.75)	3.46	0.97 to 5.96	0.01
**Vee_cw_ (L)**	20.81 (6.65)	17.94 (3.85)	2.87	–2.37 to 8.12	0.24		22.23 (6.28)	18.84 (3.85)	3.40	0.91 to 5.88	0.01
***f* (bpm)**	19 (3)^††^	16 (5)	3	–1 to 7	0.16		15 (2)	14 (3)	1	–1 to 2	0.50
**Ti/Ttot**	0.38 (0.05)	0.38 (0.03)	0.05	–0.02 to 0.04	0.67		0.40 (0.03)	0.40 (0.04)	–0.01	–0.05 to 0.05	0.94

The data are presented as the means and standard deviation. 95% CI: 95% confidence interval; ALS: amyotrophic lateral sclerosis; V_cw_: chest wall volume; V_rcp_%: percentage of the contribution of the pulmonary rib cage; V_rca_%: percentage of the contribution of the abdominal rib cage; V_ab_%: percentage of the contribution of the abdomen; Vei_cw_: chest wall end-inspiratory volume; Vee_cw_: chest wall end-expiratory volume; *f*: respiratory rate; Ti/Ttot: ratio of the inspiratory time to the total duration of the respiratory cycle. Student’s t-test was used for independent samples or the Mann-Whitney U test (Vcw) and Student’s t-test were used for paired samples.

† p=0.02 for the comparison of the ALS and control groups (sitting).

†† p=0.01 for the comparison of the ALS and control groups (sitting).

* p=0.02 for the comparison of the ALS and control groups (supine).

**Figure 1:**
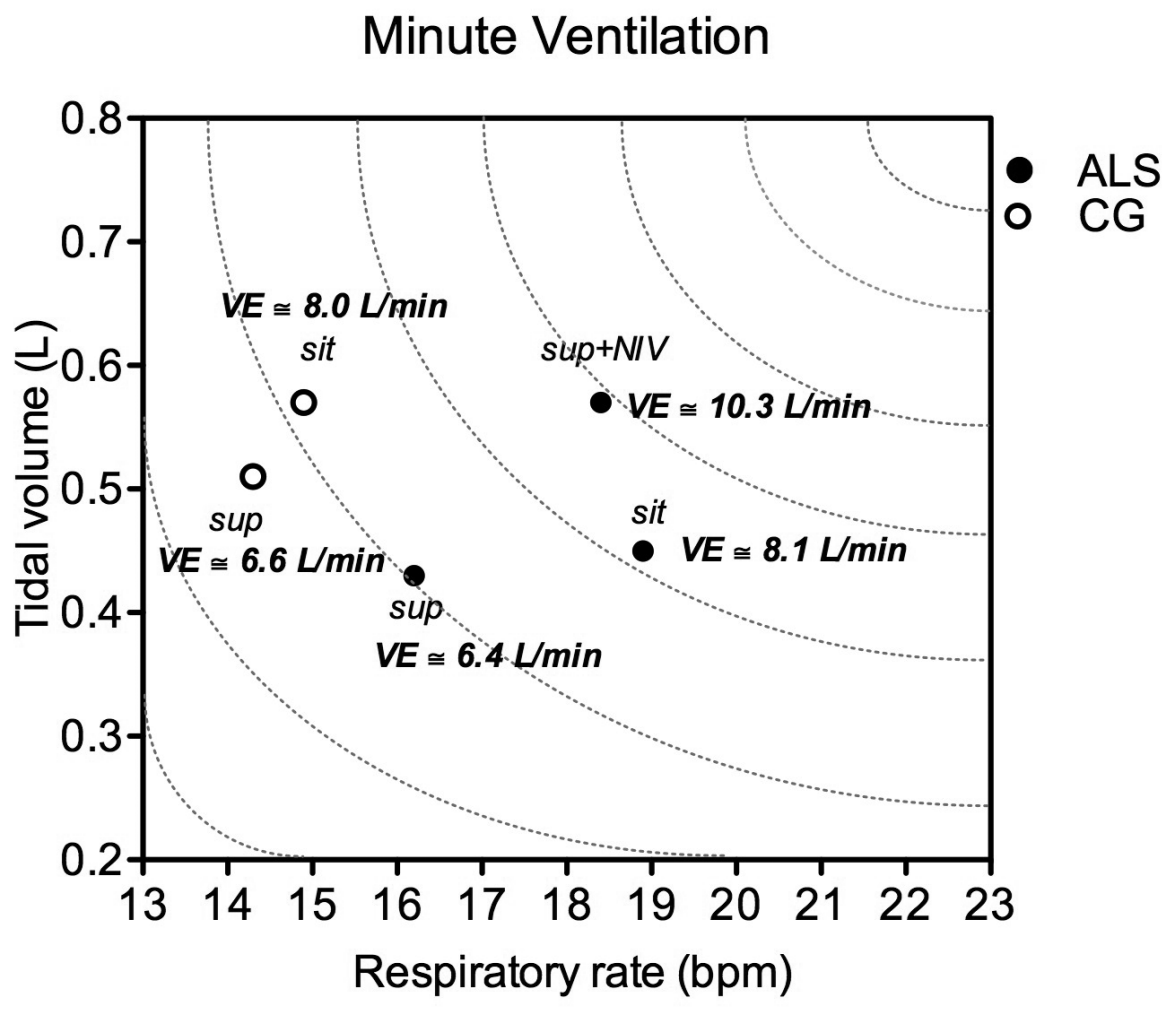
ALS: amyotrophic lateral sclerosis; VE: minute ventilation; CG: control group; NIV: non-invasive ventilation; sit: sitting position; sup: supine position without NIV; sup-NIV: supine position with NIV.

Figure 1 shows the VE data for the supine and sitting positions in the ALS and control groups, as well as in the supine position with the use of NIV for patients with ALS. During NIV, the ALS group showed a significant increase in the VE compared with that without NIV due to the increases in the V_cw_ with no significant changes observed in *f* (Table 2). No significant difference was found between the ALS and control groups, 8.13 L/min *versus* 8.01 L/min (p=0.87) in the sitting position and 6.44 L/min *versus* 6.63 L/min (p=0.82) in the supine position. However, the sitting VE of the ALS group was associated with a significantly higher *f* and a lower V_cw_ than in the control group (Table 3).

Chest wall asynchrony was analyzed (phase angle, phase relation during inspiration, and phase relation during expiration) between the pulmonary rib cage and abdominal rib cage and between the abdominal rib cage and abdomen in patients with ALS with and without NIV. No significant differences were found among the analyzed variables.

**Figure 2:**
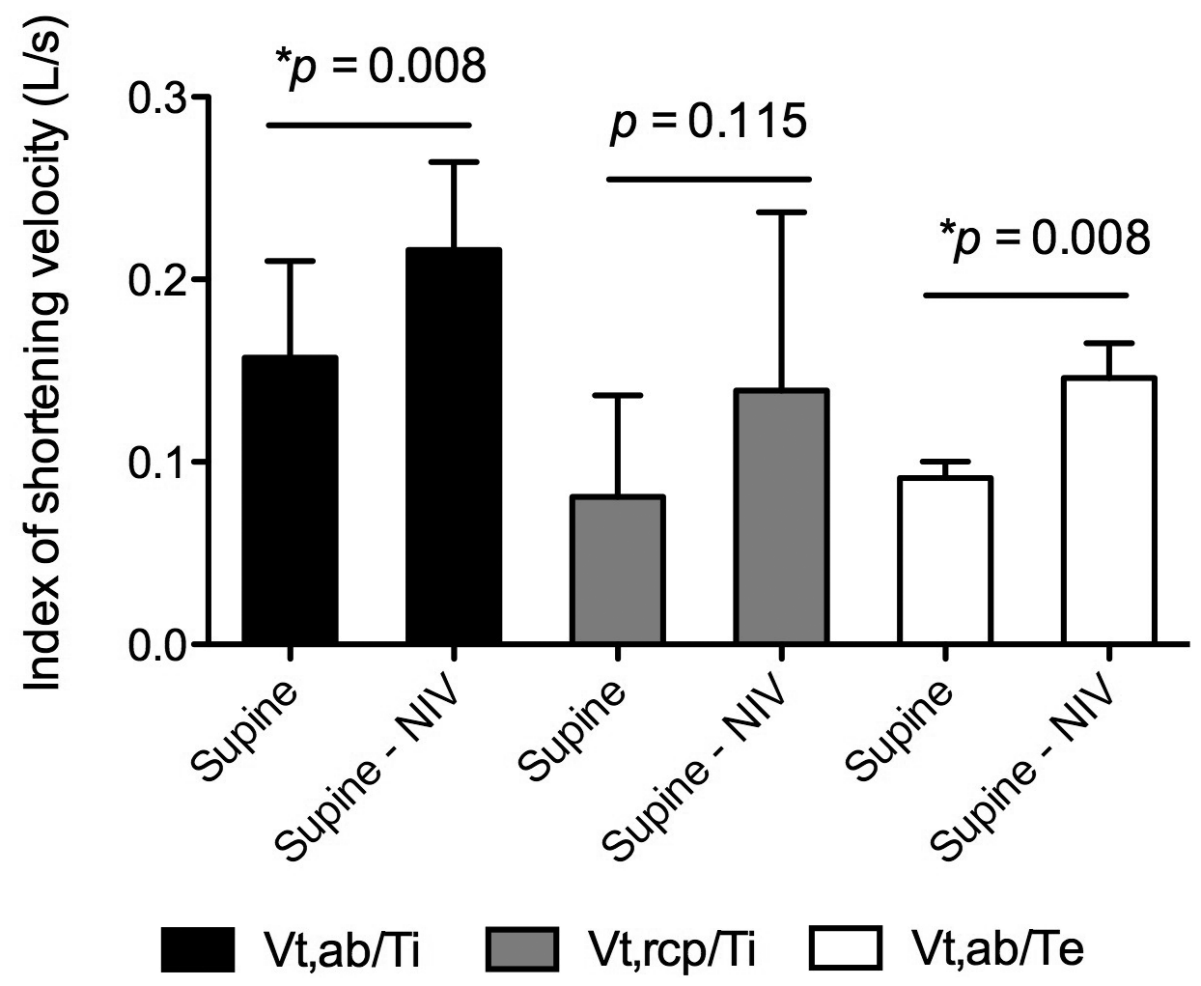
NIV: non-invasive ventilation; supine - NIV: supine position with NIV; Vt,ab/Ti: index of shortening velocity of the diaphragm muscle; Vt,rcp/Ti: index of the shortening velocity of the inspiratory muscles of the pulmonary rib cage; Vt,ab/Te: index of the shortening velocity of the abdominal muscles.

Figure 2 shows the index of the shortening velocity of the diaphragm muscle, the index of the shortening velocity of the inspiratory muscles of the pulmonary rib cage, and the index of the shortening velocity of the abdominal muscles in patients with ALS in the supine position with and without NIV. There was a significant increase in the diaphragmatic velocity and abdominal muscle shortening when the patients were using NIV.

## Discussion

The main findings of this study were that NIV promoted a significant increase in the operating volumes (chest wall end-inspiratory volume and chest wall end-expiratory volume), tidal volume of the chest wall, minute ventilation, and in the index of the shortening velocity of the diaphragm and abdominal muscles in patients with ALS without bulbar dysfunction. Moreover, patients with ALS presented a significantly lower percentage of contribution of the abdomen compared with the control group in the supine position. To our knowledge, this is the first study to assess the effects of NIV and posture on the chest wall volumes of patients with ALS. It is important to note the precision and accuracy of the instrument used to assess these parameters (OEP)^24^.

In this study, the acute response to NIV was a significant increase in the V_cw_ and VE in patients with ALS. NIV optimizes the alveolar ventilation in individuals with restrictive ventilatory defects^18^ ^,^ ^25^. The increases in tidal volume and minute ventilation have already been described in patients with neuromuscular disease^25^. However, to our knowledge, there have been no previous studies that have investigated the effects of NIV on the breathing pattern of patients with ALS, so there is no basis for comparison with our findings. During NIV, significant increases in the diaphragmatic velocity and abdominal muscle shortening were also seen. These findings could indicate that these muscles work more efficiently during NIV, contributing to the increase in the pulmonary volumes^26^.

This pioneering study demonstrated that the chest wall end-expiratory volume increased during NIV, which could contribute to preserving the peripheral airway patency and to more adequate hematosis. Furthermore, there were no significant differences in the percentage contributions of each of the three chest wall compartments when NIV was implemented. These data indicate that there was no significant change that might be interpreted as a loss or gain in relation to ventilation/perfusion.

With regards to *f* and Ti/Ttot, no significant differences were observed with the use of NIV. Because the spontaneous/timed mode was used with a back-up frequency of 14 bpm, the patients maintained control over their respiratory frequency and rhythm. To our knowledge, there have been no previous studies that have investigated the effects of NIV delivered in the spontaneous/timed mode on the *f* and Ti/Tot in patients with ALS or other neuromuscular diseases. However, similar results were found for *f* in healthy individuals under NIV used in spontaneous mode^27^.

Compared with the control group in the supine position, the lower contribution of the abdominal compartment in the ALS group was similar to that shown in the study by Lo Mauro et al.^9^ conducted in patients with DMD. In patients with NMDs, diaphragmatic dysfunction is initially observed in the supine position. A larger contribution of the rib cage compartment could suggest greater use of the inspiratory muscles of the rib cage^6^. Considering that the majority of patients assessed in this study presented with functional independence and none used a wheelchair, diaphragmatic involvement could be considered an early stage of dysfunction. OEP assessment of the chest wall motion has been shown to be sensitive in detecting these differences.

One important finding of the study was the higher percentage contribution of the abdominal rib cage in the supine position of patients with ALS compared with healthy individuals. It can be hypothesized that, in the ALS group, deficient diaphragm motion led to a significant increase in the abdominal rib cage; however, this was not enough to increase the percentage contribution of the abdomen.

Patients with ALS presented with a higher *f* in the sitting position compared with the control group, which could be explained by the restrictive characteristics of this disease. Additionally, our data show that despite the fact that there was no significant difference in the VE in the sitting or supine positions between patients with ALS and the controls, the VE in the supine position was associated with a significantly higher *f* in patients with ALS, which might suggest that they have a less efficient breathing pattern (Figure 1 and Table 2)^9^.

Another important finding was the significant reduction in the contribution of the rib cage compartment (based on the Vrcp% and Vrca%) and the significant increase in the contribution of the abdominal compartment (Vab%) in the supine position compared with the sitting position in both groups (Table 3). These results are in accordance with earlier studies, where the body position significantly affected the kinematics of the chest wall of healthy individuals and NMD patients^10^ ^,^ ^28^. A study conducted by Romei et al.^28^ that evaluated the effects of posture on the thoracoabdominal movement in healthy subjects observed similar results. The increase in the abdominal contribution in the supine position can be explained by the elastic properties of the rib cage and abdomen. According to Agostoni and Rahn^29^, when patients are at rest in the sitting position, the abdomen exhibits compliance similar to that of the rib cage, whereas in the supine position, only the abdomen changes these elastic properties and increases in compliance, so the pattern is related to chest wall physiology.

The asynchrony of the chest wall in both groups seems to be similar to that in healthy individuals in a previous study^30^; however, the present study analyzed three compartments of the chest wall by OEP instead of two compartments by respiratory inductance plethysmography. Nevertheless, the most interesting aspect of these data is the fact that NIV did not trigger a significant increase in asynchrony, indicating good adaptation in ALS patients.

One of the limitations of this study was that it did not provide a greater period of adaptation for the patients with ALS receiving NIV because a period of prior ventilation might have positively affected the results. However, no significant difference was observed in the asynchrony variables with NIV use. The absence of reference values in healthy individuals must also be highlighted. However, we accounted for this by performing the comparison with an age- and sex-matched control group. Another aspect to be considered a limitation is the small sample size (n=9). Despite the fact that this number of subjects was sufficient based on the initial sample size calculation, it is important to take into account the potential occurrence of Type II error. However, considering the lack of data in the literature, our results present novel findings in this field.

In conclusion, the results of this study showed that NIV promoted a significant increase in the chest wall volumes (tidal volume, end-inspiratory, and end-expiratory) as well as increases in the indices of the shortening velocity of the diaphragm and abdominal muscles. The clinical implications of these results are that NIV can improve the lung volumes without interfering with the compartmental distribution. Moreover, in the supine position, the patients with ALS presented a significantly lower percentage of contribution of the abdomen compared with the control group. Therefore, the OEP technique was able to reveal modifications in the breathing pattern related to ALS even in early-stage patients. OEP can be used as a new strategy to evaluate this patient population.
